# MskAge—An Epigenetic Biomarker of Musculoskeletal Age Derived From a Genetic Algorithm Islands Model

**DOI:** 10.1111/acel.70149

**Published:** 2025-06-19

**Authors:** Daniel C. Green, Louise N. Reynard, James R. Henstock, Sjur Reppe, Kaare Gautvik, Mandy J. Peffers, Daryl P. Shanley, Peter D. Clegg, Elizabeth G. Canty‐Laird

**Affiliations:** ^1^ Department of Musculoskeletal and Ageing Science Institute of Life Course and Medical Sciences, University of Liverpool Liverpool UK; ^2^ The Medical Research Council Versus Arthritis Centre for Integrated Research Into Musculoskeletal Ageing (CIMA) University of Liverpool Liverpool UK; ^3^ Biosciences Institute Newcastle University Newcastle UK; ^4^ Department of Medical Biochemistry Oslo University Hospital Oslo Norway; ^5^ Unger‐Vetlesen Institute, Lovisenberg Diaconal Hospital Oslo Norway; ^6^ Department of Plastic and Reconstructive Surgery Oslo University Hospital Oslo Norway

**Keywords:** ageing, biomarker, epigenetics, genetic algorithm, musculoskeletal system

## Abstract

Age is a significant risk factor for functional decline and disease of the musculoskeletal system, yet few biomarkers exist to facilitate ageing research in musculoskeletal tissues. Multivariate models based on DNA methylation, termed epigenetic clocks, have shown promise as markers of biological age. However, the accuracy of existing epigenetic clocks in musculoskeletal tissues are no more, and often less accurate than a randomly sampled baseline model. We developed a highly accurate epigenetic clock, MskAge, that is specific to tissues and cells of the musculoskeletal system. MskAge was built using a penalised genetic algorithm islands model that addresses multi‐tissue clock bias. The final model was trained on the transformed principal components of CpGs selected by the genetic algorithm. We show that MskAge tracks epigenetic ageing ex vivo and in vitro. Epigenetic age estimates are rejuvenated with cellular reprogramming and are accelerated at a rate of 0.45 years per population doubling. MskAge explains more variance associated with in vitro ageing of fibroblasts than the purpose‐developed skin and blood clock. The precision of MskAge and its ability to capture perturbations in biological ageing make it a promising research tool for musculoskeletal and ageing biologists.

## Graphical abstract

Created in BioRender. Laird, E. (2025) https://BioRender.com/bqfajrn.

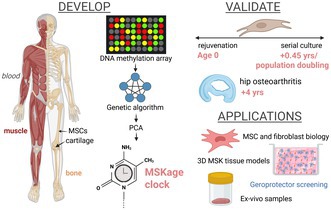

## 1 Introduction

Ageing is a complex and multifaceted process manifesting in a progressive decline of cellular function that is historically linked to the passing of time (Kirkwood 2005). While societal norms associate an individual's age with the number of years since birth, the rate of organismal development and decline is non‐linear across a lifespan and varies between tissues and organisms. This non‐linearity and heterogeneity differentiate biological ageing trajectories from those typically associated with chronological ageing. Owing to the complexity of the ageing process, accurate biomarkers of biological age are becoming increasingly valuable in research and clinical settings (Lu et al. 2019). Most existing biomarkers primarily focus on accessible tissues, such as blood, skin and saliva (Horvath et al. 2018; Levine et al. 2018; Lu et al. 2019). Although these biomarkers are useful for investigating ageing rates at the population level, they may not be as applicable to all body tissues, such as those in the musculoskeletal system. To maximise the likelihood of successfully increasing health span and lifespan, interventions will likely need to rejuvenate cells throughout the entire organism. Consequently, identifying biomarkers that facilitate ex vivo research on musculoskeletal tissues has the potential to elucidate anti‐ageing interventions currently beyond the scope of existing biomarkers.

In the past decade, DNA methylation (DNAm) clocks have emerged as some of the most promising and accurate biomarkers of age (Hannum et al. 2013; Horvath 2013a; Levine et al. 2018; Lu et al. 2019; Voisin et al. 2020). DNAm clocks are multivariate statistical or machine learning models that predict chronological age or age‐related outcomes based on the proportion of methyl groups (CH_3_) present on DNA at an optimised selection of CpGs. Notably, CpGs selected by clocks are not necessarily those within ageing genes but rather those with associations to age in thousands of samples (Horvath 2013b). The relative stability of DNA methylation allows DNAm clocks to achieve higher accuracy compared to biomarkers developed on other layers of molecular data (Xia et al. 2021). Moreover, the addition of a methyl group to cytosine by DNA methyl transferases (DNMTs) is reversible via its subsequent hydroxylation by the Ten Eleven Translocase (TET) enzymes, providing a dynamic mechanism that can be exploited to track and potentially modify the rate of biological ageing through the methylome (Wu and Zhang 2017). The divergence between predicted epigenetic age and chronological age is referred to as epigenetic age acceleration (Quach et al. 2017). It has been well established that DNAm clocks can predict age acceleration rates independently of chronological age, age‐related disease, and age‐related functional outcomes (e.g., hand grip strength), making them promising candidates as biomarkers of ageing (Horvath 2013b; Horvath et al. 2018; Levine et al. 2018; Lu et al. 2019; Quach et al. 2017).

Ageing profoundly impacts musculoskeletal function, leading to the deterioration of bone, cartilage and muscle, which can cause diseases such as osteoporosis and osteoarthritis, as well as the ageing syndrome of frailty (Roberts et al. 2016). Although attempts have been made to define biomarkers of ageing in musculoskeletal tissues, these primarily focus on biomarkers of bone turnover, skeletal muscle mass, or assessments of physical activity (Kemp et al. 2018). While these biomarkers are useful for in vivo monitoring of physical function, they do not directly assess the age of musculoskeletal cells and cannot be applied ex vivo. To facilitate ex vivo research on musculoskeletal tissues, highly accurate and specific epigenetic biomarkers or other molecular clocks are likely required.

Some existing epigenetic clocks, such as Horvath's original multi‐tissue clock, are applicable to a wide range of tissue and cell types (Horvath 2013b). However, the representation of musculoskeletal tissues in the training of these epigenetic clocks is limited compared to other tissues, making them less reliable for musculoskeletal ageing research due to the tissue‐specific nature of DNA methylation. The primary drawback of non‐specific epigenetic clocks is their poor accuracy and high variance in musculoskeletal tissues, which has implications for designing experiments with appropriate power. Obtaining musculoskeletal samples for research often involves highly invasive procedures, such as surgical operations or post‐mortem extractions, highlighting the need for more accurate and specific epigenetic clocks to design appropriately powered studies. Recently, a highly accurate epigenetic clock (error +/− 4.6 years) was developed to track the age of human skeletal muscle using ~19,000 CpGs common between the Infinium 27 k, Infinium 450 k and Infinium EPIC arrays (Voisin et al. 2020). The skeletal muscle clock exemplifies an instance where the limitations of Horvath's original multi‐tissue clock were addressed, particularly its poor calibration for underrepresented samples in the training set of the model.

In the present study, we demonstrate that existing epigenetic clocks are no more and, in some instances, less accurate than a randomly sampled baseline model across multiple musculoskeletal tissues. We employed a genetic algorithm‐based meta‐heuristic feature selection framework to construct a musculoskeletal tissue‐specific epigenetic clock, optimising CpG selection to minimise error within each tissue. Recently, it was shown that the algebraic transformation of CpG methylation into principal components for use as features in training an epigenetic clock addresses several technical issues arising from CpG feature models (Higgins‐Chen et al. 2022). Our final model, MskAge, uses a modified version of this framework, where principal component analysis is calculated after feature selection with the genetic algorithm. We demonstrate that this modified approach, applied to our dataset, improves accuracy compared to calculating principal components of the entire feature space, resulting in a highly accurate epigenetic clock (error +/− 3.51 years) across all musculoskeletal tissues and blood samples in the dataset. Although MskAge is designed to predict chronological age, it resets across multiple independent cellular rejuvenation datasets and strongly correlates with population doubling in vitro. A tutorial for calculating MskAge can be found on Github: https://github.com/laird‐lab/Msk‐Age.

## 2 Results

### 2.1 Acquired DNA Methylation Datasets

**FIGURE 1:**
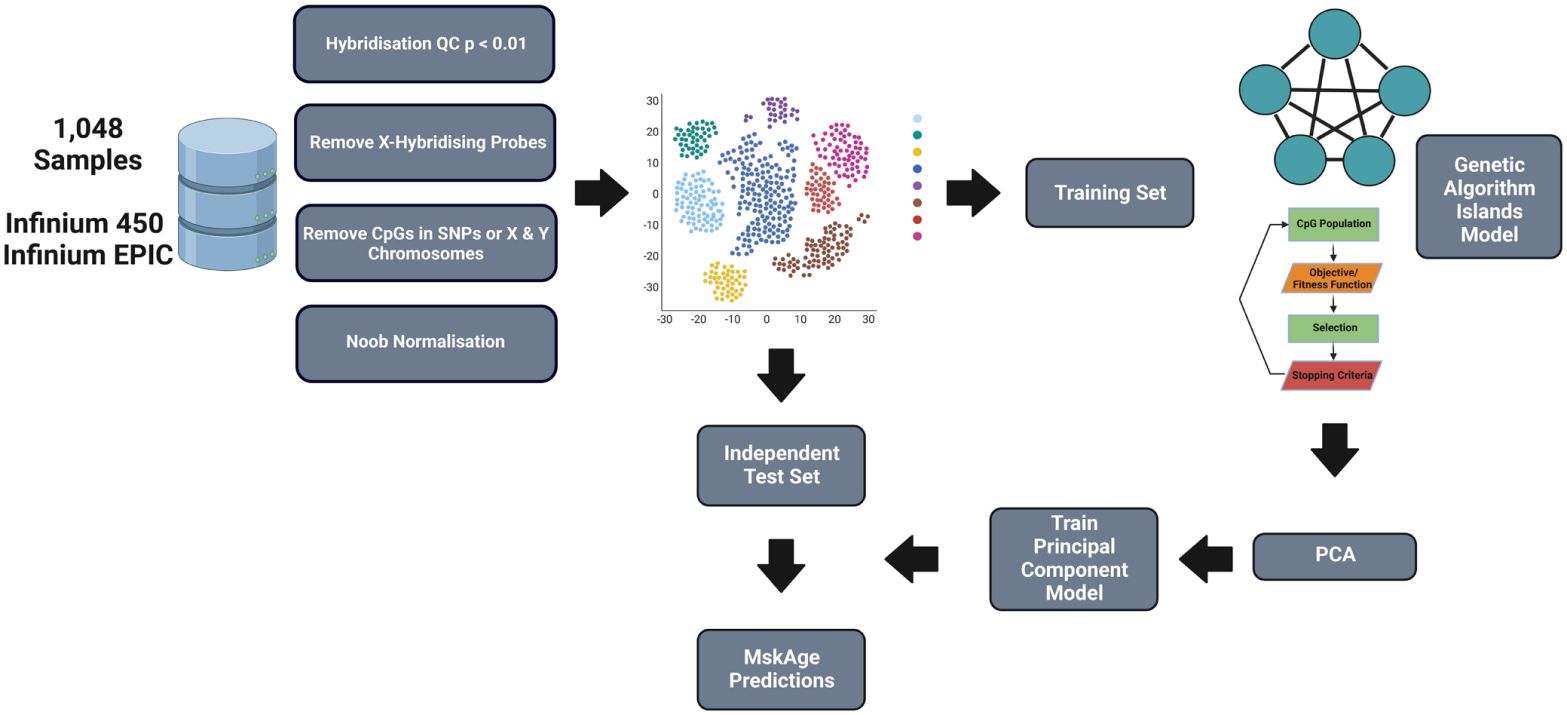
Schematic of analysis workflow for the development of Musculoskeletal Age (MskAge). The schematic depicts the collated samples undergoing quality control and filtering, being merged into a matrix and finally overviews the process of training the genetic algorithm and principal component model.

Infinium 450 k and EPIC methylation array data were acquired from searches of the public domain repository Gene Expression Omnibus (GEO) and novel data generated within the UK Center for Integrated Musculoskeletal Ageing (CIMA). A total of 1048 samples were identified across both platforms. Figure 1 shows an overview of the analysis pipeline for the generation of MskAge.

### 2.2 Methods of Quantile Normalisation Skew Epigenetic Age Estimates

With a plethora of normalisation methods available for methylation array technologies, the impact of preprocessing on epigenetic age estimates is an important consideration (Aryee et al. 2014; Fortin et al. 2017; Teschendorff et al. 2013). To explore this issue and guide the choice of normalisation for MskAge development, we computed the epigenetic age of 16 cartilage samples with a relatively narrow age range (50–60 years) using different methods. Our analysis revealed that for PhenoAge, GrimAge and Hannum clocks, the Quantile normalisation employed by both Quantile and Funnorm methods significantly affected epigenetic age estimates (Figure S1). This effect is further highlighted by a correlation matrix computed for each respective normalisation method, demonstrating that Quantile normalisation methods produce distinct methylation profiles that cluster independently from non‐quantile methods (Figure S2). These methods lead to significantly exacerbated errors in epigenetic clocks not trained on quantile‐normalised data (FDR < 0.001) (Figure S1). Based on these results, we select single‐sample normal exponential using out‐of‐band probes (ssNoob) as our chosen method of normalisation, but reasoned that with the exception of Quantile and Funnorm, other normalisation methods would be applicable to MskAge.

### 2.3 Existing Epigenetic Clocks Are Inaccurate in Musculoskeletal Tissues Relative to a Bootstrapped Randomly Samples CpG Model

**FIGURE 2:**
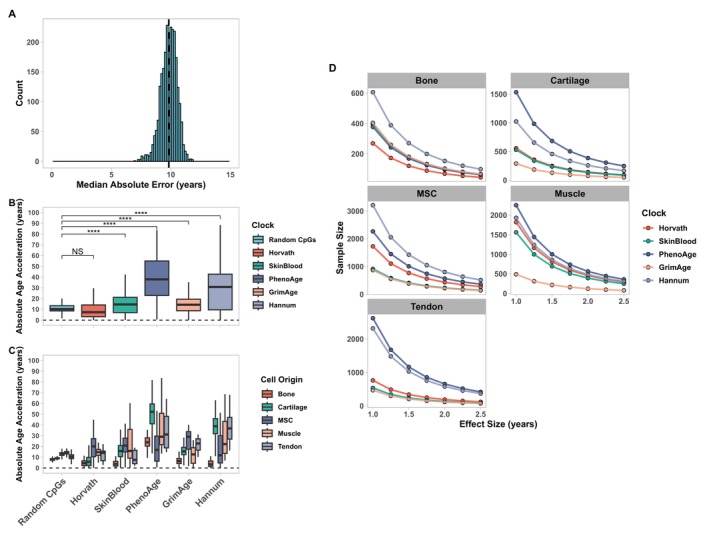
A random CpG model outperforms existing epigenetic clocks in musculoskeletal tissues. (A) Histogram displaying the distribution of median absolute errors (MAE) from 2500 iterations of an elastic net regression model containing 350 randomly sampled CpGs evaluated on an independent test dataset; the dashed vertical line represents the median MAE (+/− 9.83 years). (B) Boxplots of absolute age acceleration derived from existing epigenetic clocks and the errors from the randomly sampled models. Boxplots display the combined errors across all musculoskeletal tissues (B) and the specific errors for each musculoskeletal tissue (C). Boxplots display median errors and the interquartile range. Units are expressed in years. (D) Power calculations derived from respective errors of each existing epigenetic clock for a range of given effect sizes 1–2.5 years; the y axis refers to the sample size required to achieve 80% power for each respective effect size.

We assessed the feasibility of using existing epigenetic clocks as age biomarkers in cartilage, bone, mesenchymal stromal cells (MSCs), skeletal muscle and tendon tissue. To draw a relative comparison, we compared errors of existing clocks to a ‘randomly sampled’ model, which ran 2500 elastic net regression models each on 350 randomly sampled CpGs from the training dataset and evaluated the predictions they made on independent test data. The distribution of the errors can be seen in Figure 2A, with a median absolute error of +/− 9.83 years for all tissues indicated by the dashed line. We compared the randomly sampled model errors and errors from existing epigenetic clocks averaged across all musculoskeletal tissues (Figure 2B) and split by each musculoskeletal tissue (Figure 2C). No existing epigenetic clock performs significantly better in terms of error than the randomly sampled model (Table 1). The Horvath clock had comparable performance with no statistically significant difference between errors (*p* = 0.31), all other epigenetic clocks had significantly greater errors than the randomly sampled model (*p* < 0.05) (Table 1).

**TABLE 1:** Confidence intervals (CI) and false discovery rate (FDR) from pairwise Student's *t*‐tests comparing errors from existing epigenetic clocks to a model containing randomly sampled CpGs.

Epigenetic clock	95% CI	False discovery rate	Significance
Horvath	−1.06 to 0.34	0.31	NS
SkinBlood	4.52–6.35	7.7e−28	****
PhenoAge	27.12–30.37	5.8e−135	****
GrimAge	4.34–5.84	2.7e−34	****
Hannum	16.42–19.55	8.6e−78	****

*Note:* ****p < 0.0001.

### 2.4 Errors Associated With Existing Epigenetic Clocks Reduce the Capacity to Detect Perturbations in Epigenetic Ageing in Musculoskeletal Tissues

To further quantify the impact of errors from existing epigenetic clocks, we simulated power calculations for theoretical experiments. Power calculations were simulated based on detecting an effect size difference in that experiment of 1–2.5 years at 80% power and a 5% false positive rate. Using existing epigenetic clocks, we demonstrate that the consequences of high errors diminish the feasibility of designing appropriately powered in vitro experiments across most musculoskeletal tissues tested (Figure 2D). One example is that of MSCs, a commonly used cell type in tissue engineering that has attracted many efforts that attempt to characterise and modulate aspects of biological age (Al‐Azab et al. 2022). Reliably detecting an effect size of 2.5 years in MSCs would require > 200 samples using any of the existing epigenetic clocks.

### 2.5 Development of a Highly Accurate Principal Component‐Based Clock in Musculoskeletal Tissues

**FIGURE 3:**
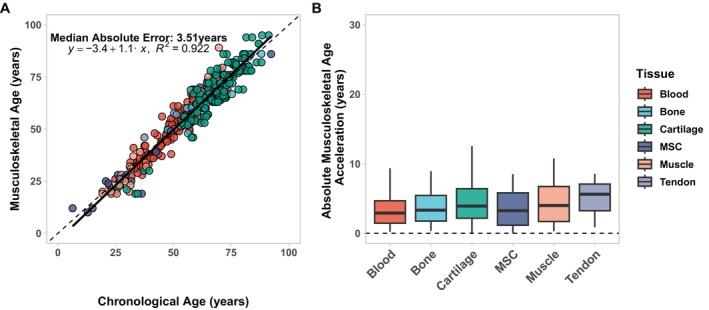
MskAge is a highly accurate predictor of age in musculoskeletal tissues and blood. (A) Linear regression of predicted epigenetic age generated with MskAge (*y* axis) vs. chronological age (*x* axis) on independent test data (*n* = 307). Points are colored by tissue origin. (B) Boxplot of Median Absolute Errors (MAE) of epigenetic age acceleration and chronological age from predictions generated with MskAge on independent test data (*n* = 307) split by tissue origin. Boxplots display median errors and the interquartile range. Units are expressed in years.

To build MskAge, we collected data from the public domain and within CIMA of 1048 Infinium Methylation 450 k or Infinium Methylation EPIC array samples that comprised multiple musculoskeletal tissues and blood. To eliminate bias, model development was performed on a training set (70%) and evaluation of error on an independent test set (30%). A penalised genetic algorithm islands model was designed to perform feature selection. MskAge was the result of regressing the principal components on the CpGs selected by the genetic algorithm against a transformed version of chronological age. The median absolute error across all tissues in the independent test data was +/− 3.51 years. The *R* ^2^ of MskAge regressed against chronological age in the test set was 0.92 (Figure 3A), higher than that of the other clocks tested (Figure S3). With the exception of tendon, which had a median absolute error of 5.52 years, all other tissues displayed a median absolute error of less than 4 years (Figure 3B). Moreover, we demonstrate that MskAge errors are consistent across tissue types when expressed as a percentage of chronological age (Figure S4). The errors for each tissue are displayed in Table S1. Sample size calculations demonstrate that an effect size of 2.5 years can be achieved with less than 60 samples for any of the tissues (Figure S5), a substantial improvement over the comparator clocks (Figure 2D).

**FIGURE 4:**
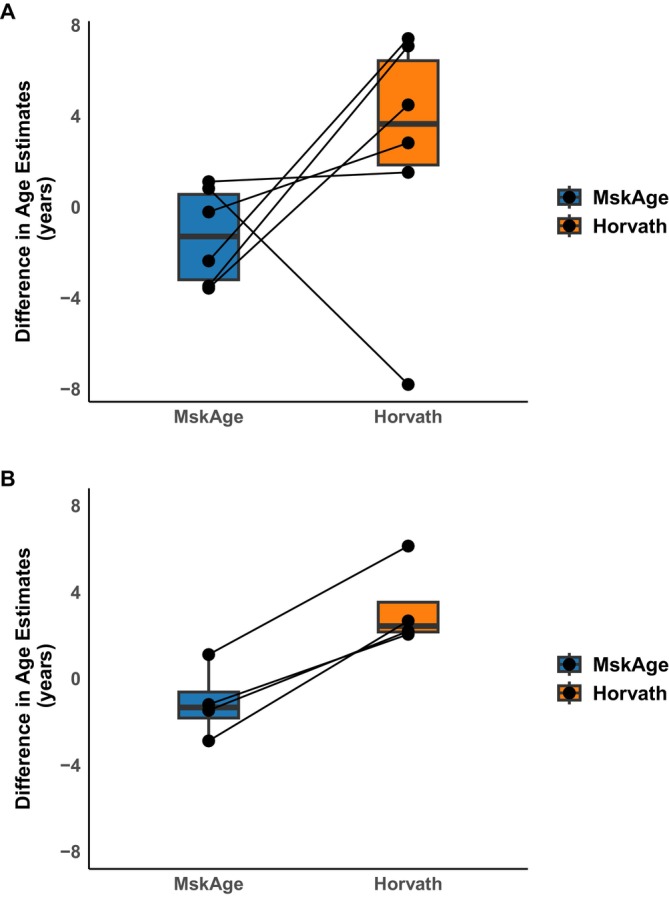
Evaluation of the performance of MSKage (3365 CpGs) and Horvath's original clock (353 CpGs) on technical replicates as a difference in age estimates. (A) Comparison of age predictions for gDNA from preserved control hip cartilage (*n* = 6) run on the Infinium 450 k array and EPIC array v1. (B) Comparison of age predictions for gDNA from MSCs (*n* = 4) run on the same EPICv1 methylation array chip.

To assess whether the PCA‐based approach reduced technical noise inherent in methylation data (Sugden et al. 2020), technical reproducibility was assessed for MSKage and Horvath's original multi‐tissue clock. When comparing 450 k and EPIC v1 array technologies, MskAge had a median difference in age estimates of −1.28 years, whereas Horvath had a difference of 3.66 years (Figure 4A). Comparing within array‐technology reproducibility for EPIC v1, MskAge had a median difference in age estimates of −1.32 years, whereas Horvath had a change of 2.43 years (Figure 4B). The tighter age predictions for MSKage demonstrate the increased reliability of MSKage on technical replicates.

To assess whether MSKage is affected by musculoskeletal cell‐type specific methylation, the epigenetic age of MSCs differentiated to osteoblasts, chondrocytes, and tenocytes (Peffers et al. 2016) was assessed (Figure S6). MskAge was not significantly affected by cell‐type shifts (*p* = 0.872), suggesting that, at least within the musculoskeletal lineage in vitro, MskAge is robust to variations in cellular composition.

### 2.6 CpGs Used to Construct MskAge Are Significantly Enriched for Terms Related to Skeletal and Mesenchyme Development

To further understand the biological context of methylation changes being captured by MskAge, we performed enrichment analysis for the genes that the CpGs in the model were located within. Using a network‐based approach which connects nodes (significant enrichment terms, FDR < 0.05) via edges (gene‐set similarity), we identify 4 clusters of Gene Ontology based enrichment terms (Figure S7). Notably, the largest cluster contained terms specifically related to mesenchyme and muscle development. We employed the same approach using the Kyoto Encylopedia of Genes and Genomes (KEGG). Significant KEGG terms (FDR < 0.05) included those in cAMP, Hippo, Wnt and calcium signalling pathways and pluripotency of stem cells (Figure S8).

### 2.7 The Musculoskeletal Clock Is Reset With Cellular Reprogramming of MSCs and Fibroblasts

**FIGURE 5:**
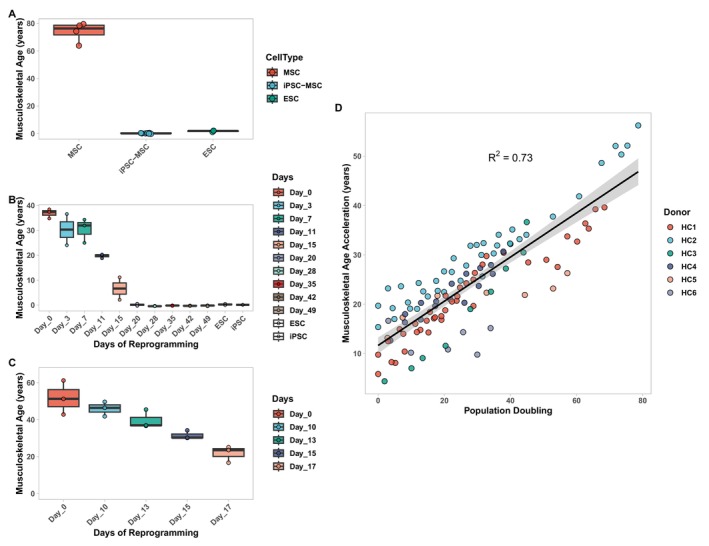
MskAge tracks cellular ageing and rejuvenation in vitro. (A) Boxplot of predicted epigenetic ages generated with MskAge of MSC donors and the same cells transformed to iPSC‐MSCs (Frobel et al. 2014). Epigenetic age predictions were also computed on ESCs from the same dataset as a positive control. (B) Boxplots of predicted epigenetic ages generated with MskAge on human dermal fibroblasts over a longitudinal experiment of reprogramming to iPSCs (Ohnuki et al. 2014). Epigenetic age predictions were also computed on ESCs from the same dataset as a positive control. (C) Boxplots of predicted epigenetic ages generated with MskAge on human dermal fibroblasts following a longitudinal experiment of iPSC reprogramming only to the maturation phase (Gill et al. 2022). (D) Linear regression of predicted epigenetic age generated with MskAge (*y* axis) versus population doubling of fibroblasts from 6 healthy donors (*x* axis) (Sturm et al. 2022).

Given that our outcome variable was a transformed version of chronological age, a question that remains is whether or not MskAge is a highly accurate predictor of chronological age, or whether the biomarker has the capacity to track biological age. We utilised three in vitro models of cellular reprogramming to address this (Frobel et al. 2014; Gill et al. 2022; Ohnuki et al. 2014). We demonstrate that reprogramming of MSCs to iPSC‐MSCs resets MskAge to approximately 0, consistent with the age observed in Embryonic Stem Cells (ESCs) (Figure 5A). Moreover, we observe the same age reduction over a longitudinal time course of fibroblasts being reprogrammed to iPSCs (Figure 5B). Importantly, MskAge was not trained on fibroblasts, but still has the capacity to detect their rejuvenation as they are programmed towards iPSCs. Likewise, for ESCs, MskAge has not observed the specific methylation profile of an ESC but detects methylation in ESCs to have an epigenetic age of 0. Finally, we computed MskAge predictions on fibroblasts that were not fully reprogrammed to iPSCs but instead only reprogrammed until the maturation phase, a point at which the cell still retains its cellular identity and can re‐differentiate (Gill et al. 2022) (Figure 5C). We observe that when fibroblasts are only partially reprogrammed, MskAge does not reset to 0 but instead decreases by approximately 30 years, which is in agreement with effect sizes observed by the Skin and Blood clock (Gill et al. 2022). It is widely accepted that cellular reprogramming is one of the most potent methods of cellular rejuvenation, thus highlighting the capacity for MskAge to track underlying changes in biological ageing.

### 2.8 Musculoskeletal Age Tracks In Vitro Cellular Ageing

We envision that one of the prominent use cases for MskAge will be to facilitate in vitro research on musculoskeletal cells. Thus, a key attribute of such a tool would be that it tracks age‐related methylated changes in vitro as well as it does on ex vivo tissues. We sought to test whether MskAge tracks epigenetic ageing in vitro over multiple cell divisions across a longitudinal in vitro time course. We reprocessed and analysed the Cellular Lifespan multi‐omics longitudinal fibroblast ageing study (Sturm et al. 2022) that includes six healthy control fibroblast cell donors cultured for up to 80 population doublings. We calculated MskAge and extracted epigenetic age predictions of existing clocks on the same data. MskAge acceleration exhibits the strongest linear relationship (*R* ^2^ = 0.73, FDR < 2.37e‐34) with population doublings relative to all clocks tested (Table 2), including the fibroblast‐specific SkinBlood clock (R2 = 0.51, FDR < 1.99e‐15) (Figure 5D). According to MskAge in this study, in vitro ageing occurs at an average rate of 0.45 years per population doubling (Table 2). The ability of MskAge to detect in vitro age acceleration as a product of population doubling is advantageous for its use in facilitating the identification of perturbations that can attenuate or accelerate the ageing process in musculoskeletal cells.

**TABLE 2:** Output of linear regression models computed from epigenetic age predictions in MskAge and epigenetic clocks in the longitudinal cellular lifespan study.

Clock	*R* ^2^	*p*	FDR	Intercept	Slope
MskAge	0.73	2.15e‐35	2.37E‐34	11.64	0.45
Horvath	0.05	0.031	0.124	18.84	0.17
SkinBlood	0.51	1.99e−15	1.79e−14	−7.27	0.33
PhenoAge	0.05	0.033	0.124	3.09	0.2
GrimAge	0.03	0.079	0.158	26.82	−0.04
Hannum	0.66	6.90e−23	6.9e−22	−36.4	0.41
PCHorvath	0.18	2.58e−05	2.0e−4	23.08	0.14
PCSkinBlood	0.11	0.001	0.005	11.37	0.16
PCPhenoAge	0.4	1.25e−11	1.0e−10	59.21	0.27
PCGrimAge	0.02	0.165	0.166	52.75	0.03
PCHannum	0.39	3.77e−11	2.64e−10	36.77	0.22

Abbreviation: PC, principal component version of the clocks.

### 2.9 Musculoskeletal Age Is Accelerated in Lesioned Osteoarthritic Cartilage

**FIGURE 6:**
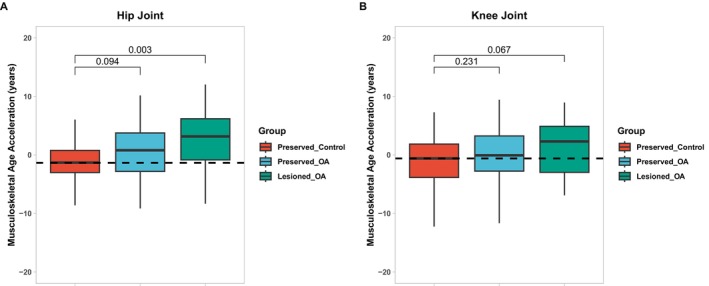
MskAge is accelerated in lesioned OA cartilage. (A) Predicted MskAge of Preserved Control (*n* = 73), Preserved OA (*n* = 39) and Lesioned OA (*n* = 26) Hip cartilage samples. (B) Predicted MskAge of Preserved Control (*n* = 58), Preserved OA (*n* = 85) and Lesioned OA (*n* = 17) Knee cartilage samples.

MskAge was trained on non‐lesioned cartilage samples. To test whether MskAge is accelerated in the context of osteoarthiritis (OA) we reprocessed 298 hip and knee non‐OA and OA cartilage samples. Lesioned cartilage exhibits significant Musculoskeletal Age acceleration in the hip (4.029 years, FDR = 0.003) and borderline significant Musculoskeletal Age acceleration in the knee (3.16 years, FDR = 0.067) relative to preserved non‐OA controls (Figure 6A,B; Table S2). The correlation between MSKage and chronological age was also assessed (Figure S9) and was strong in both hip and knee, though it is not optimal given both control and OA samples are present. Interestingly, cartilage extracted from non‐lesioned hip OA patients still exhibits a trend towards being epigenetically older than preserved control hip cartilage (2.056 years, *p* = 0.094) (Figure 6A; Table S2). While the direction of change is also the same for preserved OA knee cartilage relative to preserved control knee cartilage (1.02 years) the difference is non‐significant (*p* = 0.231). In contrast, Horvath's clock detected no differences in age acceleration between cartilage samples (Figure S10).

It is noteworthy that no significant differences in MskAge were observed when we compared healthy to osteopenic and osteoporotic bone samples (Figure S11) or with exercise intervention in muscle (Figure S12).

## 3 Discussion

The musculoskeletal system is crucial to the ageing process, and maintaining its proper function is key for extending both health span and lifespan in humans (Roberts et al. 2016). Although there has been extensive research on the ageing of the musculoskeletal system, few reliable biomarkers are available for musculoskeletal tissues (Kemp et al. 2018). In this study, we introduce MskAge, an accurate principal component‐based epigenetic clock that can be applied ex vivo and in vivo to investigate the epigenetic age of musculoskeletal tissues and cells. MskAge tracks biological age acceleration and reversal, making it a promising tool for studying pro or anti‐ageing interventions in musculoskeletal tissues and cells.

Recent work has emphasised the importance of preprocessing pipelines for epigenetic age prediction (Ori et al. 2022). In light of this, we evaluated important technical considerations during the construction of MskAge by assessing the impact of various array‐based normalisation methods on epigenetic age predictions. A previous study demonstrated that Horvath's age prediction is robust to the choice of data preprocessing methods (McEwen et al. 2018). While our results align with McEwen and colleagues, the same does not hold true for other clocks, particularly when employing quantile‐based normalisation methods for PhenoAge, GrimAge and Hannum. Quantile normalisation methods force data distributions to be equal, resulting in a stringent method of statistical normalisation. Notably, PhenoAge, GrimAge and Hannum clocks did not have quantile normalised data in their training sets, which may account for the exacerbated prediction errors. As a result, we recommend normalising data using ssNoob prior to making predictions with MskAge.

A common debate in the ageing biomarker literature concerns the absolute importance of accuracy when the outcome variable is chronological age (Bell et al. 2019; Zhang et al. 2019). It is indeed valid that outside of forensic settings, establishing a model that provides a perfect readout of chronological age is seldom useful. Zhang and colleagues show experimentally that as a blood‐based epigenetic clock's predictions approach near‐perfect chronological age accuracy, the clock loses its association with mortality risk, which is a proxy of biological age (Zhang et al. 2019). These findings create some subjectivity and uncertainty around the ideal level of accuracy for a biomarker to be useful. To address this question objectively, we used a two‐fold data‐driven approach to assess the applicability of existing epigenetic clocks were applicable to musculoskeletal tissues. First, we created a ‘Random CpG’ model, involving 2500 iterations of randomly selecting 350 CpGs from the 338,185 CpGs in the dataset, training a model on 70% of the data and evaluating its error on the 30% held‐out test set. We selected 350 CpGs because it is approximately equal to the mean number of CpGs in the existing epigenetic clocks tested. Remarkably, the errors from the “Random CpG” model were as accurate or, in most cases, more accurate than epigenetic age predictions using existing clocks on the same samples (Figure 2B,C; Table 1). This outcome underlines the extent to which existing epigenetic clock CpGs lack specificity for musculoskeletal tissues. A plausible explanation for the accuracy of the Random CpG model is that it reflects the degree to which ageing impacts methylation patterns across a substantial portion of the epigenome, which has been reported to be up to 20% (Porter et al. 2021). Indeed, it has been shown that accumulating stochastic variation can predict chronological and biological age (Meyer and Schumacher 2024). Accurate models of chronological age prediction have also been built using only a small number of CpGs, such as those in the genes ELOVL2, FHL2, KLF14 and TRIM59 (Woźniak et al. 2021). Secondly, we simulated power calculations based on the errors of existing epigenetic clocks across a range of theoretical effect sizes for future experiments. We propose that one of the primary applications for an epigenetic biomarker of age in musculoskeletal tissues would be to identify compounds that could perturb the ageing process in vitro. According to the power calculations, designing adequately powered studies using existing epigenetic biomarkers would be largely impractical and unfeasible both logistically and financially. The combined evaluation of both above methods objectively underscores that existing epigenetic clocks are not suitable for quantifying epigenetic ageing in musculoskeletal tissues. As a result, we developed MskAge with the goal of providing substantial improvements in predictive accuracy and musculoskeletal‐tissue specificity compared to other epigenetic clocks. Furthermore, we benchmarked MskAge to show that its enhanced age prediction accuracy did not compromise its ability to detect changes in biological age.

Developing a multi‐tissue epigenetic clock can be viewed as a multi‐objective optimisation problem. DNA methylation serves as a critical factor in determining cellular identity, and as a result, differences in methylation between tissues tend to be more pronounced than the more subtle changes occurring with age (Kim and Costello 2017). The creation of a multi‐tissue epigenetic clock adds a hierarchical structure to a dataset. When age distributions across various tissue types are unbalanced, a naïve model that overlooks tissue origin might inadvertently capture tissue‐specific methylation patterns rather than age‐related changes. MskAge was developed to address this issue by employing a multi‐level genetic algorithm islands model that minimises within‐tissue age prediction errors. Genetic algorithms are meta‐heuristic frameworks that optimise outcomes based on evolutionary principles (Scrucca 2013). We utilised a penalised ridge regression fitness function within the genetic algorithm framework to efficiently search and reduce the feature space. Ridge regression models have been successfully employed as learners in fitness functions for genetic algorithms across various applications (Ahn et al. 2012; Zhang and Horvath 2005). The L2 penalty of the ridge regression cost function causes the coefficient weights of correlated features to shrink towards each other without completely removing them (Friedman et al. 2010). This coefficient shrinkage is desirable in the context of CpG methylation, which exhibits high collinearity. Furthermore, since the coefficient weights are shrunken but not removed, as in lasso or elastic net, ridge regression ensures that the inclusion of CpGs is assigned at some weight, enabling them to be penalised for their inclusion in the model. To penalise the inclusion of CpGs, we assigned each CpG a penalty of 0.01 years in the genetic algorithm, promoting a fitness benefit that encourages convergence towards a model with fewer features, provided that the removal of a specific CpG does not inflate the model error by more than the specified penalty. Rather than a single genetic algorithm, we distributed the evolutionary optimisation task across four independent islands that exchanged information on their best solutions every ten iterations. Utilising multiple islands for the optimisation task enables a more efficient search of the feature space and diminishes the likelihood of converging on local optima (Scrucca 2017; Whitley et al. 2015). After 200 iterations, our ‘fittest’ model, which includes the selection of CpGs optimised by the genetic algorithm to achieve the lowest cross‐validated accuracy, contained 3365 CpGs. Rather than further reducing this selection, we transformed all 3365 CpGs into linear principal components using singular value decomposition (SVD). The use of principal components as a dimensionality reduction method in constructing multivariate models with omics data is not new (Mishra et al. 2011). However, the first attempt using this approach in the development of epigenetic clocks was published recently (Higgins‐Chen et al. 2022). What distinguishes our method from Higgins‐Chen and colleagues is we adapted this approach by performing SVD on M values after extensive variable selection through the genetic algorithm. This approach resulted in an improved test set error of +/− 3.51 years, compared to an error of +/− 5.21 years when creating a principal component model on the full CpG matrix before variable selection.

The advantages of using principal components as features in epigenetic clocks have been discussed previously (Higgins‐Chen et al. 2022). An epigenetic clock constructed on principal components of a larger number of age‐related CpGs improves both inter‐ and intra‐dataset predictions, addressing the significant challenge of array‐related ‘batch to batch’ variability (Higgins‐Chen et al. 2022). Taking such an approach means that MskAge is optimised for data generated from Infinium 450 or Infinium EPIC arrays due to the large amount of CpGs that need to be quantified. Although it is feasible to develop a subset of MskAge coefficients that explain maximum variance with minimal redundancy, this likely would reduce the overall accuracy of the model.

A potential limitation of the application of epigenetic clocks to bulk tissue samples is the heterogeneity in DNA methylation between cell types and the potential for cell‐type composition to change with age (Teschendorff and Horvath 2025). We were not able to apply a cell‐type deconvolution algorithm for MSKage, as comprehensive methylation‐specific cellular reference sets for musculoskeletal tissues are rare. Instead, we demonstrated that MSKage acceleration in MSCs differentiated to multiple musculoskeletal cell types in vitro was similar. However, it remains plausible that age‐related shifts in immune cell populations or in the vascular system could influence readouts from ex vivo tissue samples.

A further improvement in future interactions of epigenetic clocks could include accounting for the non‐linearity of the ageing process (Shen et al. 2024) as reflected in methylation levels (Vershinina et al. 2021). We included an age transformation for samples less than 20 years old, though this term may have a negligible effect as few samples tested here were in this age range. For adult samples, quadratic terms were found to refine clock performance with CpG interaction terms, higher order polynomials, and spline‐based models cited as future directions to enhance clock performance (Bernabeu et al. 2023).

To date, little mechanistic evidence has emerged from epigenetic clock coefficients, and it has often been challenging to infer functional relevance to the ageing process from CpG‐based models containing a few hundred CpGs (Bell et al. 2019). The highly co‐linear nature of the epigenome may inflate the chances of clocks being built on methylation changes that are correlated but not causal to the ageing process. The method employed in this study does not eliminate the inclusion of correlated, non‐causal features. We found that with a large number of correlated CpGs, there was significant enrichment for Gene Ontology processes associated with development. The enriched KEGG pathways identified key signalling pathways, such as Hippo, Calcium and Wnt, which are commonly implicated in both musculoskeletal ageing and disease. Wnt signalling regulates numerous cellular functions and importantly contributes to both bone and cartilage regeneration, and its dysregulation exacerbates osteoarthritis (Houschyar et al. 2018). Additionally, Hippo signalling has been implicated in regulating skeletal muscle mass (Watt et al. 2015). Interestingly, both the significant Gene Ontology and KEGG terms contained modules related to hormonal regulation and Cushing syndrome, respectively. Cushing syndrome is a condition related to the overproduction of cortisol, and hypercortisolism is proposed to induce a premature ageing phenotype (Aulinas et al. 2013). While further research is required to delineate the functional relevance of methylation changes in these pathways, the enrichment of MskAge coefficients in musculoskeletal and ageing‐specific pathways is promising.

We assessed MskAge's capacity to quantify biological ageing methylation changes using cellular reprogramming and longitudinal in vitro ageing. Cellular reprogramming, the gold standard of experimental cellular rejuvenation, reverts somatic cells into an ESC‐like state, reversing many ageing hallmarks and rejuvenating the transcriptome and methylome (Frobel et al. 2014; Gill et al. 2022; Horvath 2013b; Lapasset et al. 2011; Ohnuki et al. 2014). We reanalysed three independent cellular reprogramming datasets, demonstrating that MskAge consistently reports the age of iPSCs and ESCs as zero and tracks the biological reversal of fibroblasts and MSCs during reprogramming, even though some of these cell types were not included in MskAge's training (Frobel et al. 2014; Gill et al. 2022; Ohnuki et al. 2014). It is plausible that by using the principal components of a large number of CpGs (3365), MskAge has the scope to capture cell‐type specific and cell‐type independent features of biological ageing. In contrast to cellular reprogramming, serially passaged primary cells without genetic perturbations undergo systemic changes recapitulating ageing and eventually become senescent (Hayflick and Moorhead 1961). MskAge exhibited a strong linear relationship with population doubling in serially passaged fibroblasts, outperforming other clocks tested, including the SkinBlood clock (Horvath et al. 2018). MskAge's ability to track in vitro ageing is advantageous for musculoskeletal ageing research, which often relies on the extraction and culture of ex vivo cells. Furthermore, MskAge ticks at an approximate rate of 0.45 years per population doubling in primary fibroblasts, enabling researchers to objectively investigate perturbations that may modulate this rate. MskAge constitutes a valuable new tool for musculoskeletal biologists and ageing researchers that is a highly accurate predictor of epigenetic age in musculoskeletal cells and tissues ex vivo and in vitro. Its superior accuracy facilitates the design of experiments with appropriate statistical power compared to existing epigenetic clocks in musculoskeletal tissues. In addition to its precision, MskAge effectively tracks the biological age of cells through well‐known ageing perturbations and provides a means by which serial passaging of cells can be used as a model of epigenetic ageing in vitro. MskAge also facilitates the monitoring of pharmacological, genetic, and nutritional interventions on musculoskeletal cells.

## 4 Methods

### 4.1 Data Acquisition

Datasets used for the development of the Musculoskeletal Clock were acquired internally from the Center for Integrated Musculoskeletal Ageing (CIMA) or the public repository Gene Expression Omnibus (GEO). GEO search terms were restricted to data generated on the Infinium Methylation 450 k or Infinium Methylation EPIC array platforms. Sources and descriptions of the acquired data are given in Table S3, and additional information is in Supporting Information methods.

### 4.2 Data Processing

All data processing was performed in R (4.1.2). Raw IDAT files and raw methylated and unmethylated intensity matrices available for 16/18 datasets were reprocessed using the functionality within the minfi package (Aryee et al. 2014). CpG probes were removed from IDAT files if their methylated and unmethylated signal intensities were not significantly above that of control probes (detection *p*‐value > 0.01). Probes were further removed if they mapped within 2 base pairs of a single nucleotide polymorphism (SNP), to the X or Y chromosome, or were found to cross‐hybridise to multiple genomic locations (Pidsley et al. 2016). The remaining 2/18 datasets were processed as above, with the exception that signal intensity relative to control probes could not be assessed.

### 4.3 Epigenetic Age Calculations for Existing Epigenetic Clocks

All epigenetic age calculations for existing epigenetic clocks presented in this manuscript were calculated using the online DNA Methylation Age calculator with the ‘Normalise Data’ option selected (Horvath 2013a). Age calculations for the in vitro fibroblast ageing data were derived directly from the Cellular Lifespan Study shiny application Cellular Lifespan Study (Sturm et al. 2022). It is valuable to note that whilst two iterations of a skeletal muscle clock have been published, they could not be evaluated herein because all of the skeletal muscle samples used in this study were used in the training sets of the two iterations of the skeletal muscle clock (Voisin et al. 2020, 2021).

### 4.4 Data Normalisation

Following the investigation of various normalisation methods, datasets used in the development of the musculoskeletal clock for which raw IDAT files were available were normalised using single sample normal‐exponential out‐of‐band (ssNoob) normalisation (Fortin et al. 2017). Datasets for which only unnormalised beta values were available were normalised using Beta Mixture Quantile Normalisation (BMIQ) (Teschendorff et al. 2013).

### 4.5 Data Imputation

Probe‐wise and sample‐wise missingness were assessed by counting row‐wise and column‐wise missing values (NA's). The data was overall complete, with > 99.5% of samples having missing values for < 1% of their CpGs. Values for the CpGs that were missing were imputed using the impute.knn function (Troyanskaya et al. 2001).

### 4.6 Defining a Random CpG Model of DNAmAge in Musculoskeletal Tissues

To establish a random CpG model, the dataset was split into training (70%) and test data (30%) with respect to tissue origin for the samples, such that 70% of each tissue was used for training within each dataset. A 10‐fold cross‐validated elastic net regression model was trained using 350 CpGs sampled at random without replacement from the 338,185 CpGs that remained after filtering and integrating the data. This process was repeated 2500 times to provide an unbiased distribution of errors (Zou and Hastie 2005). The error was calculated as the absolute Age Acceleration (DNAmAge—Age).

### 4.7 Determining Power Calculations for Given Effect Sizes

For each respective error of existing clocks in musculoskeletal tissues, power calculations were simulated based on a fixed power of 80% and significance at 5% (*p* = 0.05). Cohen's *d* was calculated as the difference in means divided by the pooled standard deviation of DNAm Age Acceleration. Power calculations were simulated for effect sizes of 1–2.5 years in increments of 0.25 years.

### 4.8 Univariate Statistical Analysis

Univariate statistical tests were computed with either pairwise Student's *t*‐tests or linear models as described in the table and figure legends. Resulting *p* values were adjusted using the Benjamini Hochberg FDR method (Benjamini and Hochberg 1995).

### 4.9 Genetic Algorithm

#### 4.9.1 Training and Test Set Splits

Data was split 70/30 into training and test sets with respect to tissue origin. Descriptions of samples within each set can be seen in Table S5.

#### 4.9.2 Feature Reduction and Selection

To reduce dimensionality by removing potentially redundant features (CpGs) we calculated the Pearson correlation coefficient of each CpG with age across each tissue in the training data and filtered out any CpGs with an absolute Pearson correlation < 0.3. After filtering, 7677 CpGs remained. We employed a penalised genetic algorithm islands model to select CpGs that were most predictive across each musculoskeletal tissue. A description of the algorithm can be found in sections below.

#### 4.9.3 Problem Description

Using the reduced set of CpGs, the aim was to develop a model that optimised the selection of CpGs for the prediction of age across multiple musculoskeletal tissues and blood that also accounted for the hierarchical nature and imbalance between tissues in the training dataset. To do this, we designed a genetic algorithm islands model as follows.

Given a set of training samples Z (Z_1_, Z_2_…Z_i_) with each Z_i_ having a corresponding chronological age A_i_, tissue original T (T_1_, T_2_…T_k_) and an associated vector of M values for CpGs S (S_1_, S_2_…S_j_). For convenience, we add a superscript index to each sample that denotes the tissue type that the sample belongs to. For example, Z_t_ denotes all samples in Z that belong to tissue T. The aim is to select a combination of S that minimises the error derived from the fitness function of predicting age A^T^ in samples Z^T^.

#### 4.9.4 Encoding the Fitness Function

To select a subset of S predictive of age across all Z^T^, each CpG S_i_ is binary encoded to a value of S^1^ or S^0^. Here we denote a chromosome C as the binary encoded selection of S for a particular iteration of the genetic algorithm, specifically (S^1^ in S). To define the fitness (accuracy) of each chromosome C, we build a ridge regression function with lambda parameter defined by 10‐fold cross‐validation in the training set. The ridge regression fitness function can be seen below.

$$\sum_{i=1}^{\left|Z\right|}{\left(y_{i}-\beta_{0}-\sum_{j=1}^{\left|C\right|}\beta_{j}x_{\mathrm{ij}}\right)}^{2}+\lambda \sum_{j=1}^{\left|C\right|}\beta_{j}^{2}$$

Using the ridge regression coefficients, 10‐fold cross‐validation on the training set Z is used to compute a mean absolute error (MAE) for each musculoskeletal tissue Z^T^. The MAE for each tissue is then averaged to calculate a single objective fitness value, which is equal to the mean MAE equally weighted for every tissue T in the training set. We penalise the inclusion of CpGs in the model by adding a weight of 0.01 years to the MAE, thus forcing the genetic algorithm to discard individual CpGs whose inclusion in the model does not reduce error by at least this amount.

$$\frac{\sum_{\mathrm{k\epsilon T}}\left(\sqrt{\sum_{\mathrm{i\epsilon}Z^{k}}\frac{{\left({\hat{y}}_{i}-y_{i}\right)}^{2}}{\left|Z^{k}\right|}}\right)}{\left|T\right|}$$

#### 4.9.5 Evolution of Chromosomes on Independent Islands

The task of minimising the fitness value by the selection of the fittest chromosomes is distributed across four different islands within the genetic algorithm. Initially, a population that contains 200 randomly sampled chromosomes is generated and split equally across eight island nodes. Each island evaluates its population of chromosomes as described above, resulting in 50 fitness values. The top 5% of chromosomes that yield the fittest individuals (i.e., those with the lowest fitness values) in each island are retained from that population. Single‐point crossover is applied to occur at a rate of 70% to the top 5% of fitness individuals in each island. Crossover produces child chromosomes that are the product of two parent chromosomes. Random mutations are permitted to occur in each of the child chromosomes probabilistically at a rate of 10%. The aforementioned process is repeated for 400 iterations, with the migration of the fittest chromosomes between islands every ten iterations. Algorithm parameters are given in Table S6.

### 4.10 Development of the Final Musculoskeletal Clock

To build the final model, we used the 3365 CpGs selected by the genetic algorithm to compute principal components. We trained an elastic net regression model on these principal components to predict a transformed version of age (Horvath 2013b). Age transformation was performed as follows.

If age < 20:

F(age) = log (age+1)—log(20 + 1).

Else:

F(age) = (age‐20)/(20 + 1).

To assess the accuracy of the model on hold‐out test data and new independent datasets, M values were first projected into the same principal component space as the training data. The resulting principal components were then used to make predictions with the model.

#### 4.10.1 Functional Enrichment

Functional enrichment of the 3365 CpGs was conducted using the clusterProfiler R package (Wu et al. 2021) with all identified genes from our filtered dataset as the background. CpGs were mapped to genes based on the Illumina manifest file. Enrichment was performed using the enrichGO function that performs enrichment of a vector of genes within Gene Ontology terms. Redundant terms were removed using the simplify function with a similarity cutoff of 0.7. Significant gene ontology pathway networks were clustered and visualised using an Enrichment Map. Edges connecting the nodes denote the overlap in genes between the pathways.

## Author Contributions

Conceptualisation: Peter D. Clegg, Elizabeth G. Canty‐Laird. Data curation: Daniel C. Green. Formal Analysis: Daniel C. Green. Funding acquisition: Peter D. Clegg, Elizabeth G. Canty‐Laird. Investigation: Daniel C. Green. Methodology: Daniel C. Green. Project administration: Elizabeth G. Canty‐Laird. Resources (data sets): Louise N. Reynard, Sjur Reppe, Kaare Gutvik, Mandy J. Peffers. Software: Daniel C. Green. Supervision: James R. Henstock, Daryl P. Shanley, Peter D. Clegg, Elizabeth G. Canty‐Laird. Visualisation: Daniel C. Green. Writing – original draft: Daniel C. Green. Writing – review and editing: Daniel C. Green, Louise N. Reynard, James R. Henstock, Sjur Reppe, Kaare Gutvik, Mandy J. Peffers, Daryl P. Shanley, Peter D. Clegg, Elizabeth G. Canty‐Laird.

## Conflicts of Interest

The authors declare no conflicts of interest.

## Supporting information


Data S1.

## Data Availability

The data are available from the corresponding author upon reasonable request.
