# Supplementary material for: MskAge—An Epigenetic Biomarker of Musculoskeletal Age Derived From a Genetic Algorithm Islands Model
Source: Aging Cell. 2025 Jun 19;24(9):e70149. doi: 10.1111/acel.70149 (PMC12419849; doi:10.1111/acel.70149)
Supplement: Supplementary file 1 — Data S1. [file ACEL-24-e70149-s001.docx]

**Supplementary Information**

**Supplementary Methods**

*Data Acquisition.* Samples used to build MskAge were acquired from a combination of internal Center for Integrated Musculoskeletal Ageing (CIMA) datasets and a search of the public data repository Gene Expression Omnibus (GEO). The search terms for GEO were restricted to include Infinium 450 and Infinium EPIC arrays only. Search criteria allowed for the inclusion of any cells or tissues that function as part of the musculoskeletal system and blood. Given the abundance of publicly available blood methylation datasets, we decided to restrict the inclusion to no more than approximately half of the samples collected. Where feasibly possible, we used only tissue samples that were not diseased, although it was not plausible to obtain a cohort that was free of other co-morbidities. For cultured cells, such as Mesenchymal Stromal Cells (MSCs), we utilised only control cells from the experiments and filtered out samples that were exposed to any pharmacological, nutritional or genetic manipulation. All skeletal muscle samples were obtained from the vastus lateralis. Two out of the three skeletal muscle datasets (GSE151407 and its related dataset GSE171140) were repeated measures designs of individuals subjected to exercise programs. We used only baseline samples from these experiments. A summary of samples can be seen in Supplementary Table 4.

**Supplementary Tables**

**Supplementary Table 1.** Median absolute errors of MskAge test set predictions for each respective tissue and cell type in the test dataset.

| **Tissue** | **Median Absolute Error** |
| --- | --- |
| Blood | 2.91 |
| Bone | 3.32 |
| Cartilage | 3.92 |
| MSC | 3.25 |
| Muscle | 3.99 |
| Tendon | 5.62 |

**Supplementary Table 2.** Output of pairwise students t-tests for comparisons of epigenetic age predictions derived from MskAge in hip and knee cartilage samples. A positive mean difference indicates epigenetic age acceleration in the subgroup listed on the right hand side of the contrast.

| **Contrast** | **P Value** | **FDR** | **Mean Difference (years)** |
| --- | --- | --- | --- |
| Preserved Control Hip vs Lesioned OA Hip | 4.70E-04 | 0.003 | 4.029 |
| Preserved OA Hip vs Lesioned OA Hip | 0.104 | 0.128 | 2.056 |
| Preserved Control Hip vs Preserved OA Hip | 0.047 | 0.094 | 1.974 |
| Preserved Control Knee vs Lesioned OA Knee | 0.022 | 0.067 | 3.16 |
| Preserved OA Knee vs Lesioned OA Knee | 0.107 | 0.128 | 2.14 |
| Preserved Control Knee vs Preserved OA Knee | 0.231 | 0.231 | 1.02 |

**Supplementary Table 3.** Data sources used to build MSKage.

| Tissue/Cell Type | Sample No | Source |
| --- | --- | --- |
| Blood | 544 | GSE128235, GSE42861 |
| Bone | 90 | GSE202067, PMID: 29514638 |
| Cartilage | 255 | GSE63695, CIMA Dataset(s) |
| Mesenchymal Stromal Cells | 69 | GSE202067, GSE52114, GSE79695, CIMA Dataset |
| Skeletal Muscle | 82 | GSE171140, GSE151407, GSE50498 |
| Tendon | 8 | CIMA Dataset |

**Supplementary Table 4.** Patient/Sample characteristics used to build MskAge.

| **CellType** | **Age** | **Males** | **Females** |
| --- | --- | --- | --- |
| Blood | 51 ± 12.4 | 180 | 364 |
| Bone | 64.4 ± 9.7 | 2 | 88 |
| Cartilage | 69 ± 13.9 | 106 | 149 |
| MSC | 39.1 ± 27.5 | 30 | 34 |
| Muscle | 40.5 ± 22.3 | 82 | 0 |
| Tendon | 40.8 ± 24 | 6 | 2 |
| CellType | Age | Males | Females |

**Supplementary Table 5.** Training and Testing for development of MskAge.

| **Set** | **Female** | **Male** | **Total** | **Age Range (years)** |
| --- | --- | --- | --- | --- |
| Training | 468 | 270 | 738 | 2 - 95 |
| Test | 169 | 136 | 305 | 10 - 95 |

**Supplementary Table 6.** Genetic Algorithm Parameters.

| **Parameter** | **Value** |
| --- | --- |
| Iterations | 400 |
| Population Size | 200 |
| Islands | 4 |
| Crossover | 0.7 |
| Crossover Method | Uniform Crossover |
| Mutation Rate | 0.1 |
| Mutation Method | Uniform Random Mutation |
| Migration | 10 |
| Migration Rate | 0.1 |

**Supplementary Figures**

**
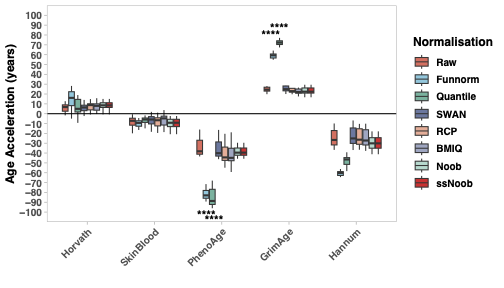
**

**Supplementary Figure 1.** Age acceleration of 16 cartilage samples (50-60 years) for Horvath, SkinBlood, PhenoAge, GrimAge and Hannum epigenetic clocks each normalised with raw (no normalisation), functional normalisation (Funnorm), quantile normalisation (Quantile), subset-within array normalisation (SWAN), regression of correlated probes normalisation (RCP), beta mixture quantile normalisation (BMIQ), normal exponential using out-of-band probes (Noob) and single-sample normal exponential using out-of-band probes (ssNoob). Units are displayed in years.

**
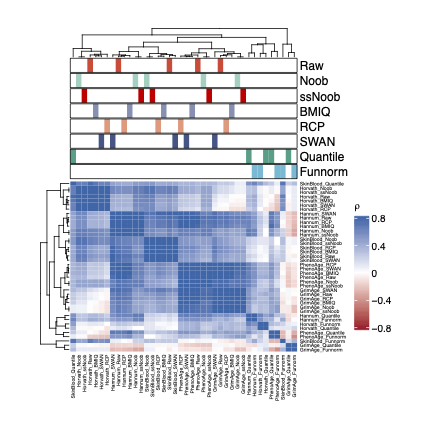
**

**Supplementary Figure 2.** Correlation heatmap of 16 cartilage samples (50-60 years) for Horvath, SkinBlood, PhenoAge, GrimAge and Hannum epigenetic clocks each normalised with raw (no normalisation), functional normalisation (Funnorm), quantile normalisation (Quantile), subset-within array normalisation (SWAN), regression of correlated probes normalisation (RCP), beta mixture quantile normalisation (BMIQ), normal exponential using out-of-band probes (Noob) and single-sample normal exponential using outof- band probes (ssNoob). Correlation is spearman’s rho. Clustering was performed using Euclidean distance with the ward.D2 linkage method.


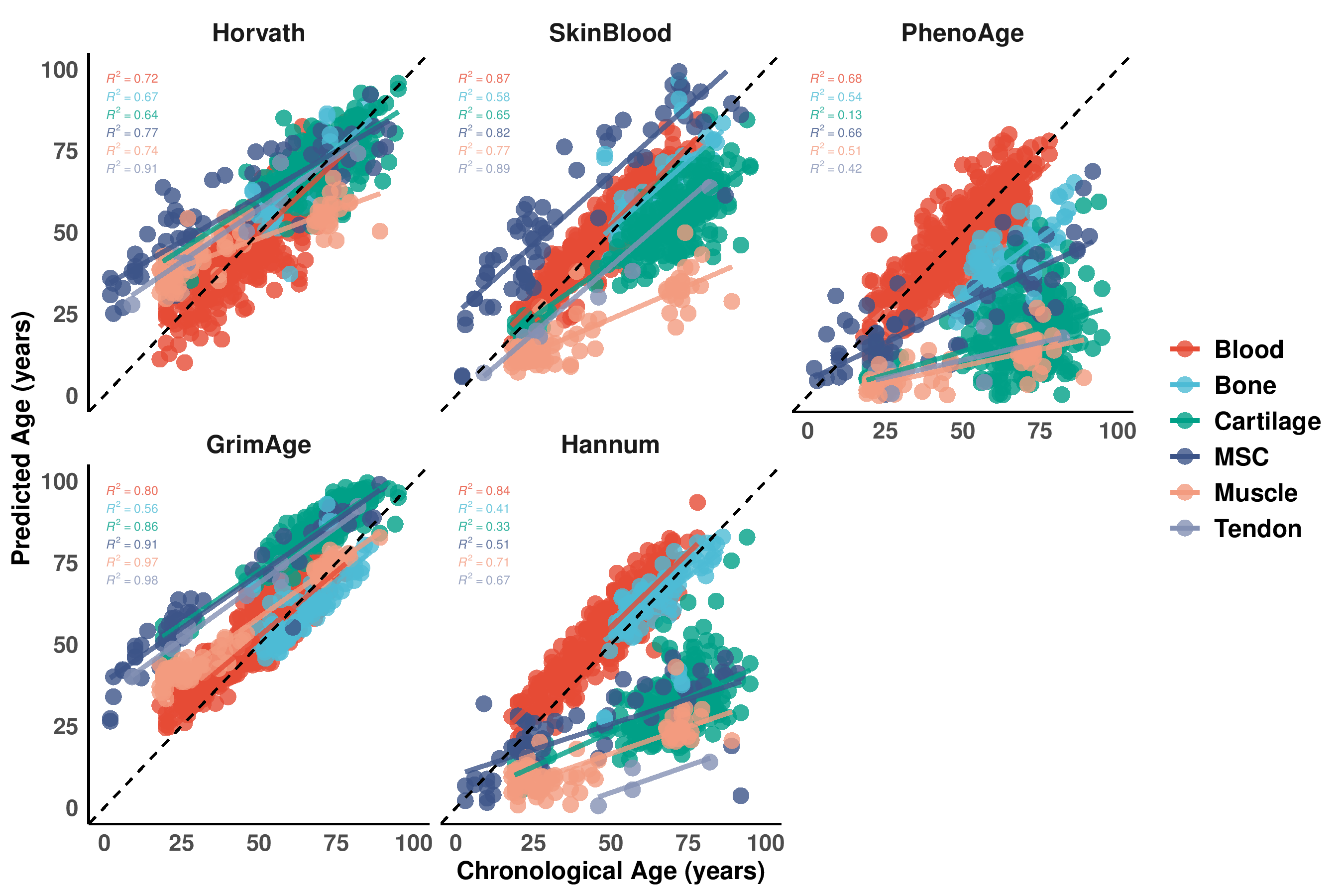


**Supplementary Figure 3.** Linear regression of predicted epigenetic age (y axis) generated with comparator clocks vs chronological age (x axis) on the independent test data (n=307). Correlation coefficients (R^2^) for each tissue type are shown in the corresponding colour.


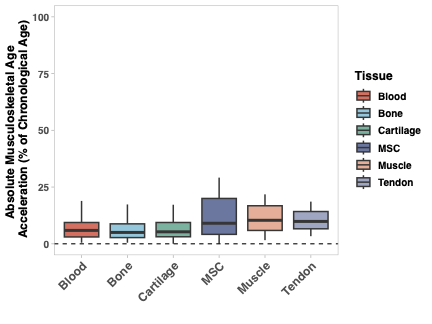


**Supplementary Figure 4.** Boxplot of Median Absolute Errors (MAE) from predictions generated with MskAge on independent test data (n=307) split by tissue origin and expressed as a percentage of chronological age.

**
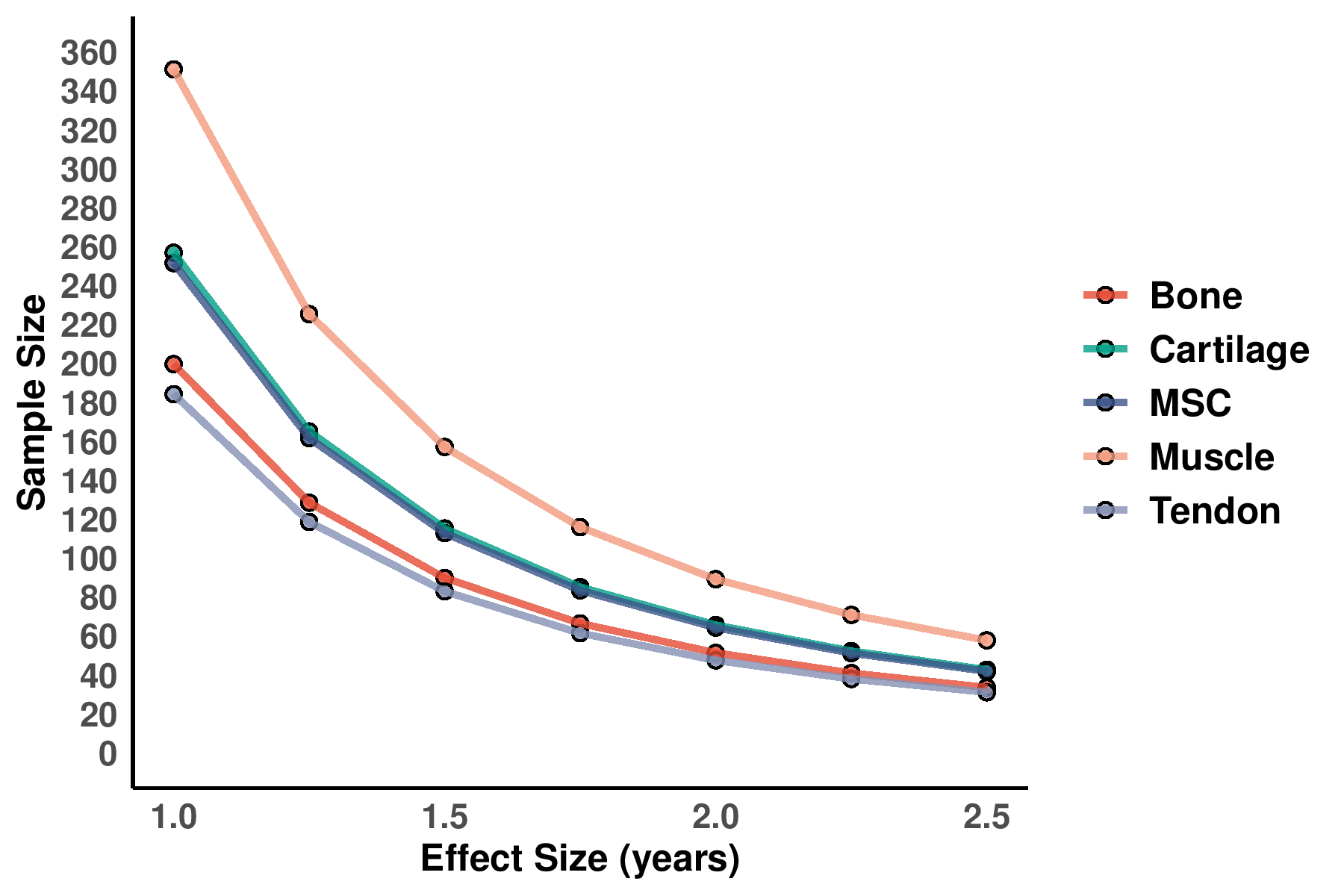
**

**Supplementary Figure 5.** Power calculations derived from the MSKage error for each tissue type for a range of given effect sizes 1 year to 2.5 years. The y axis refers to the sample size required to achieve 80% power for each respective effect size.

**Supplementary Figure 6.** MSKage acceleration determined for MSCs (n=4, ages 20-25) differentiated to chondrocytes, osteoblasts and tenocytes (Peffers, Goljanek-Whysall, et al., 2016) and profiled on the Infinium 450k methylation array. No significant differences in MSKage between cell types were detected.


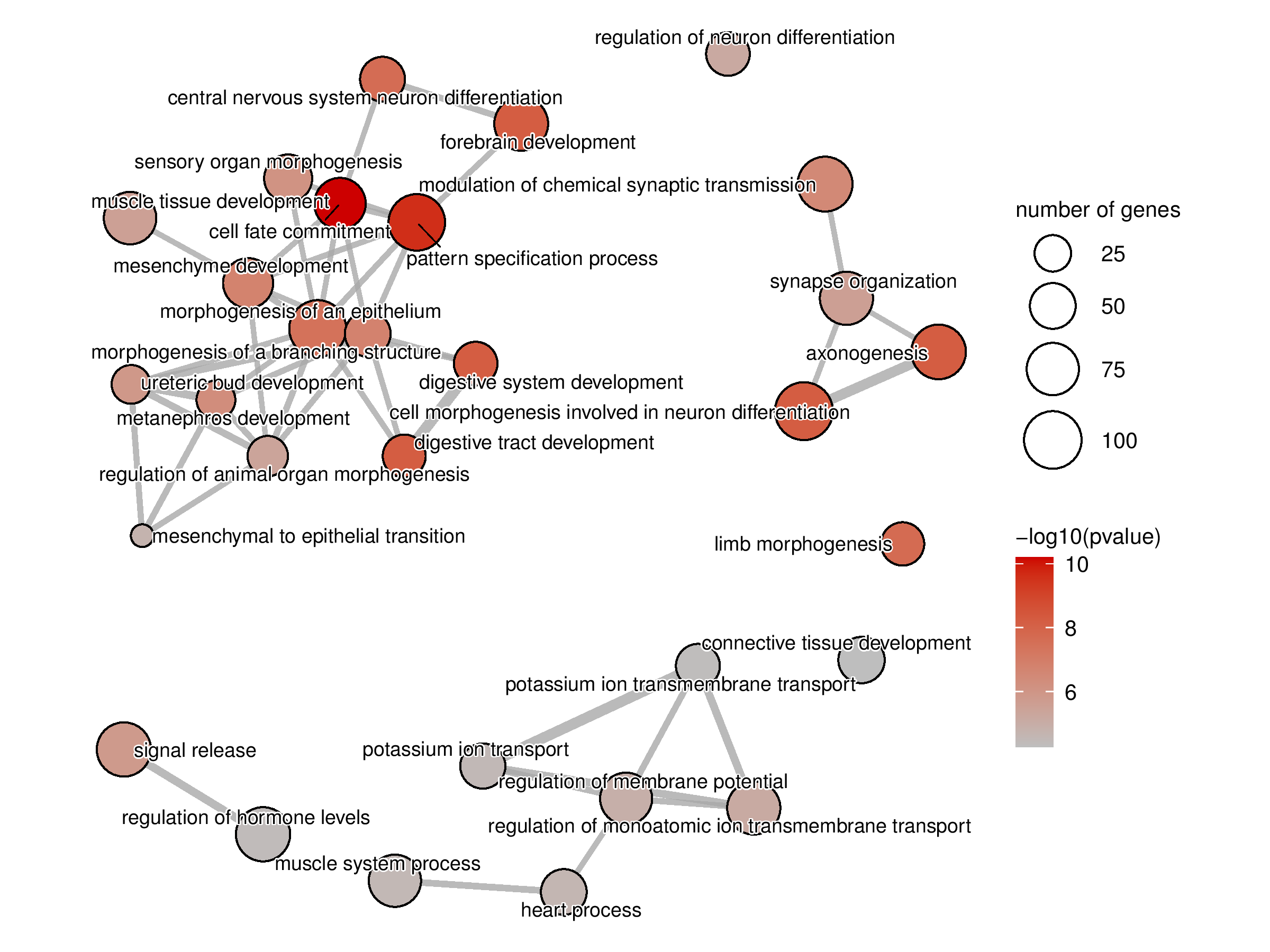


**Supplementary Figure 7. CpGs in MskAge are highly enriched for skeletal system and mesenchyme development** Enrichment map network of significantly enriched gene ontology terms for the individual coefficients of MskAge. The size of the nodes corresponds to the number of genes enriched within each gene ontology term. The colour of the node represents the -log10(FDR) of the enrichment for each term.


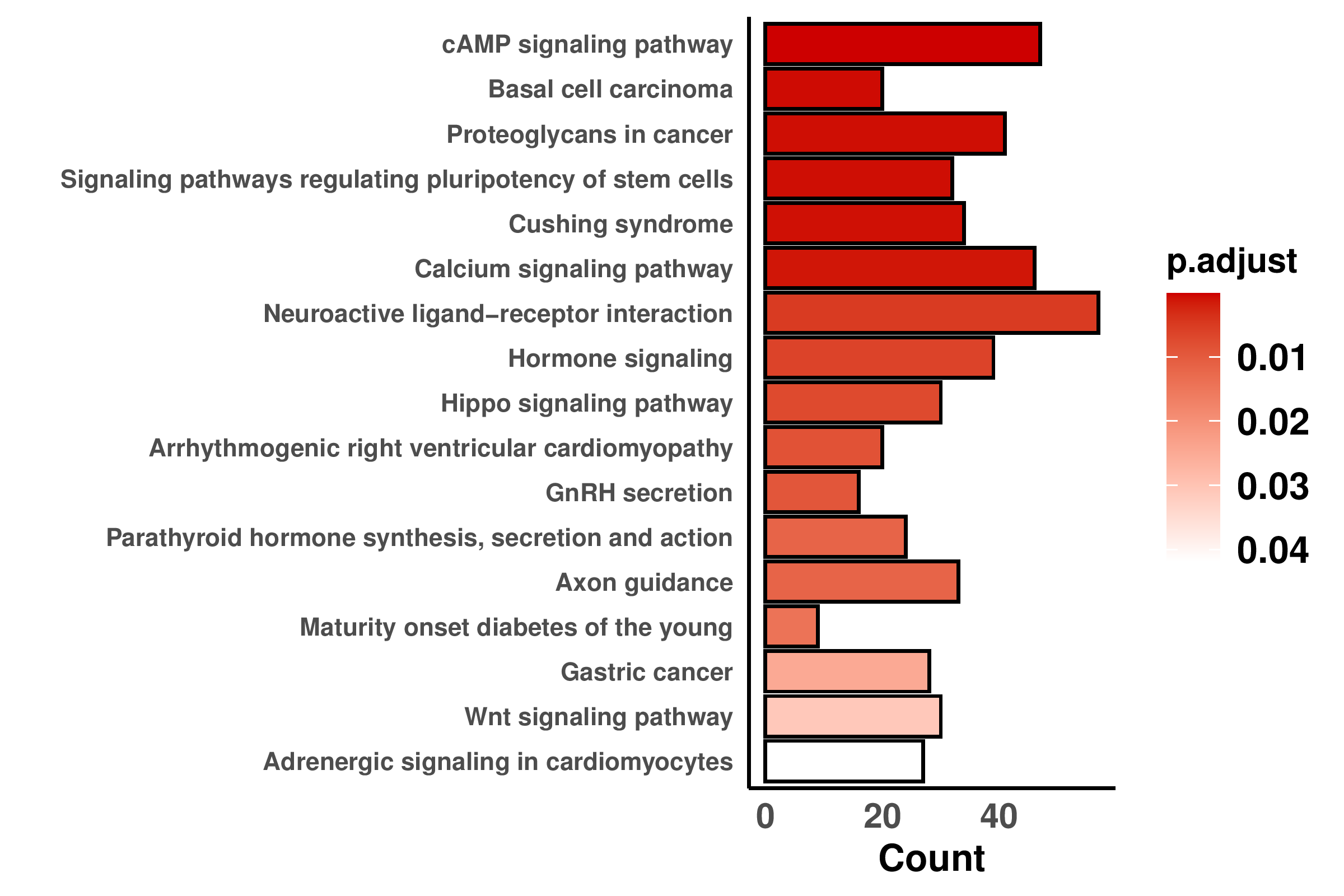


**Supplementary Figure 8.** Barplot of significantly enriched KEGG terms of genes proximal to MskAge. Bars are coloured according to their level of significance for each term. The colour of the bar represents the -log10(FDR) of the enrichment for each term.

**
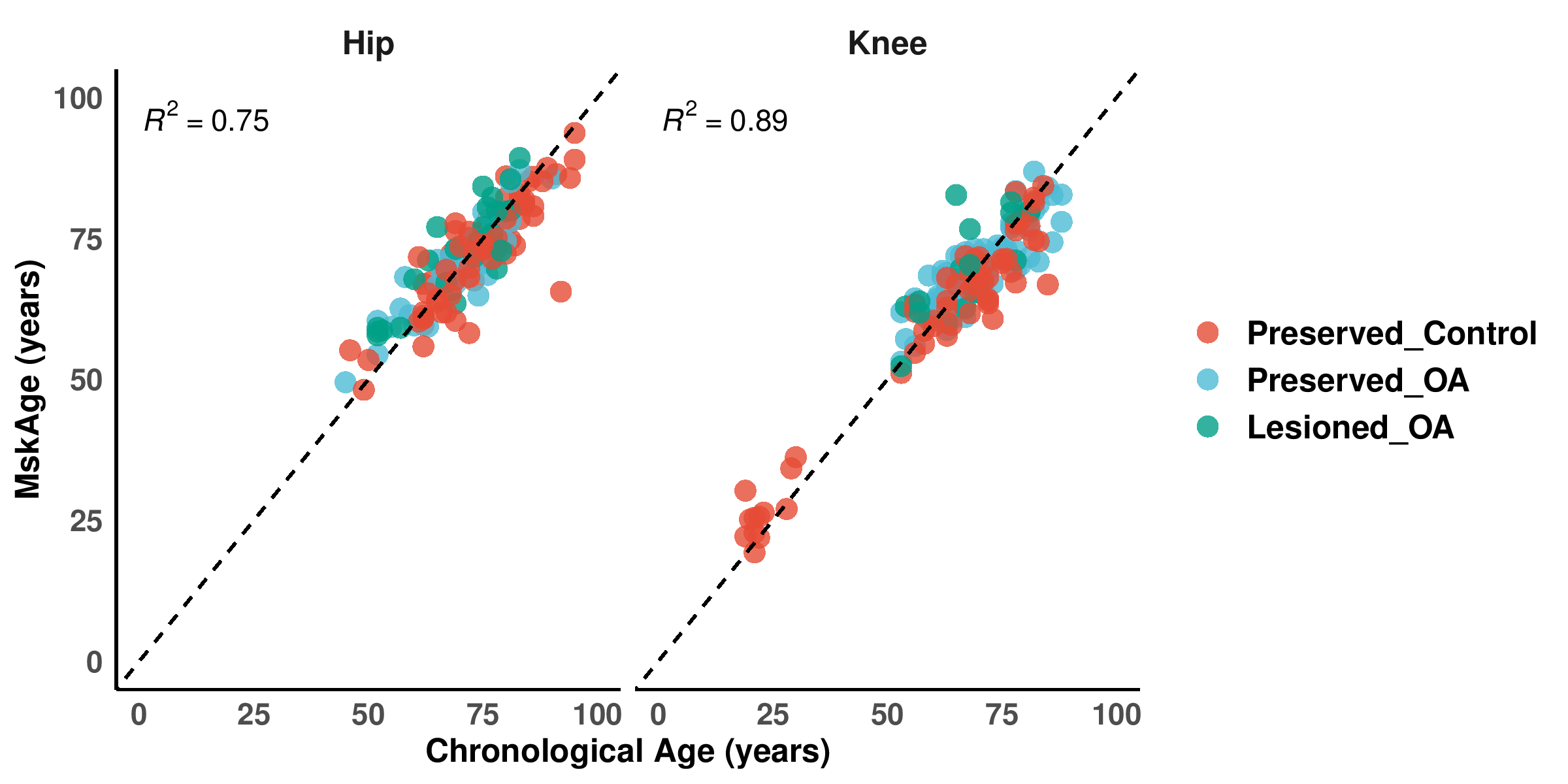
**

**Supplementary Figure 9.** Linear regression of predicted epigenetic age generated with MskAge (y axis) vs chronological age (x axis) for hip and knee cartilage samples.


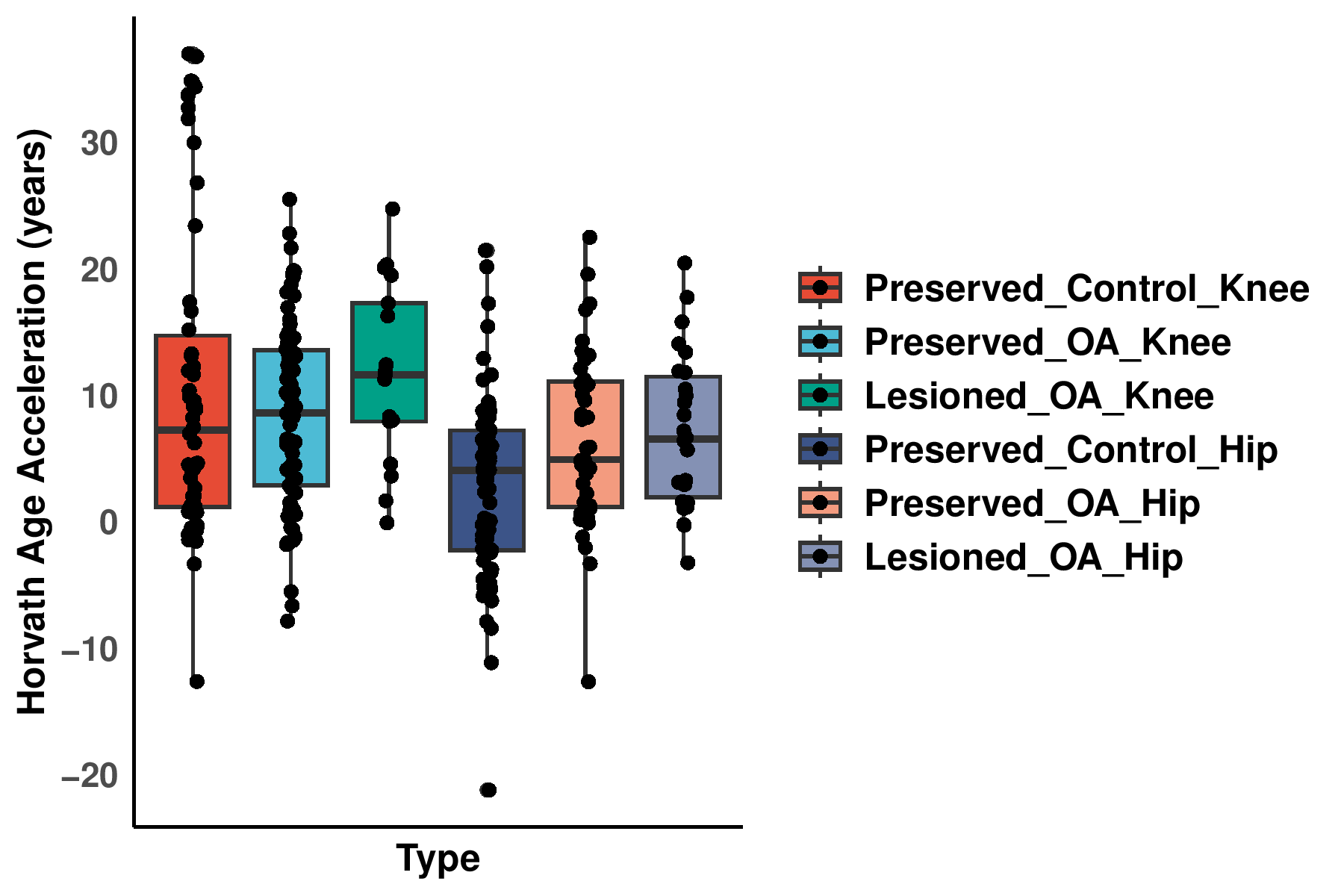


**Supplementary Figure 10.** Predicted age of cartilage samples using the Horvath clock for Preserved Control Knee (n=58), Preserved OA Knee (n=85), Lesioned OA Knee (n=17), Preserved Control Hip (n=73), Preserved OA Hip (n=39) and Lesioned OA Hip (n=26).


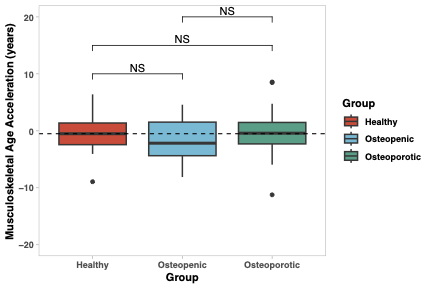


**Supplementary Figure 11.** Boxplot of Musculoskeletal Age Acceleration (years) from predictions generated with MskAge on bone biopsy samples from healthy (n=40), osteopenic (n=18) and osteoporotic (n=26) patients.


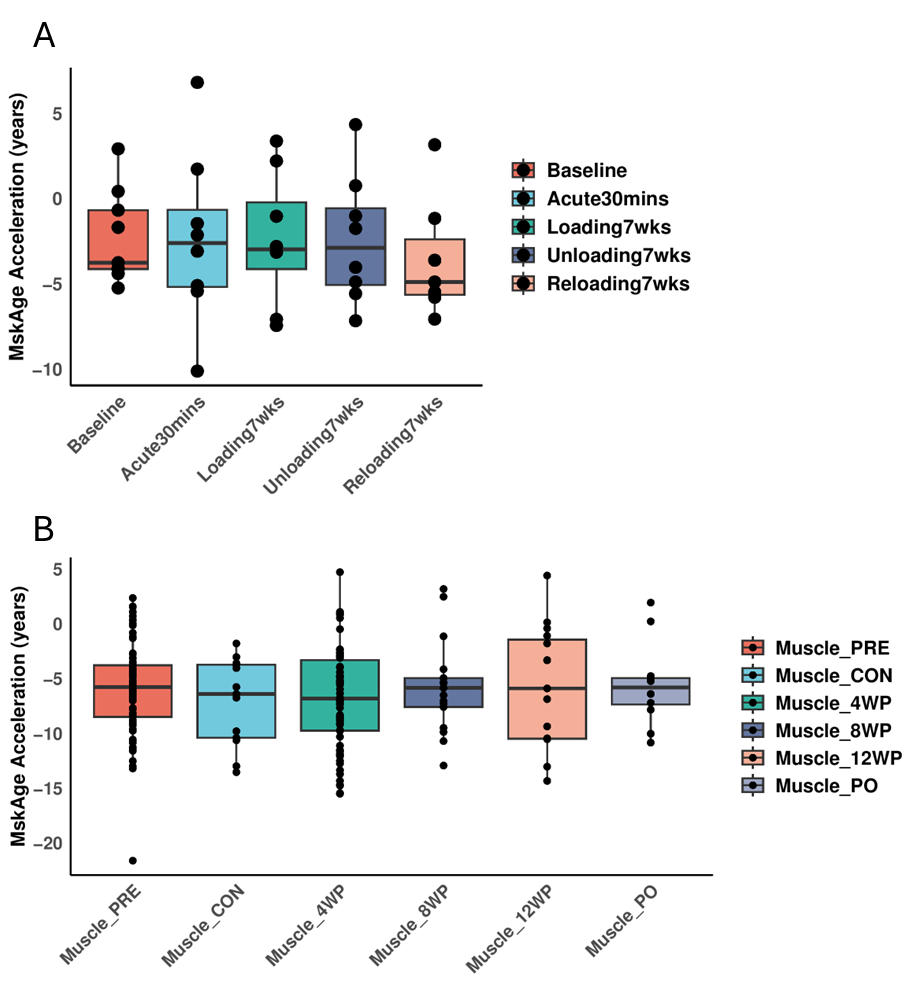


**Supplementary Figure 12.** Boxplots of Musculoskeletal Age Acceleration (years) from predictions generated with MskAge on skeletal muscle sample datasets: A (Seaborne et al., 2018), B (GSE171140). No significant differences were detected between training regimens or controls.
